# Supplementary material for: Uncovering miRNA-mRNA Regulatory Modules in Developing Xylem of Pinus massoniana via Small RNA and Degradome Sequencing
Source: Int J Mol Sci. 2021 Sep 21;22(18):10154. doi: 10.3390/ijms221810154 (PMC8472836; doi:10.3390/ijms221810154)
Supplement: Supplementary file 1 [file ijms-22-10154-s001.zip › ijms-1365557-supplementary/Additional file S2.zip!/Additional file 2/novel_29_Cluster-18410.56490_2481_TPlot.pdf]

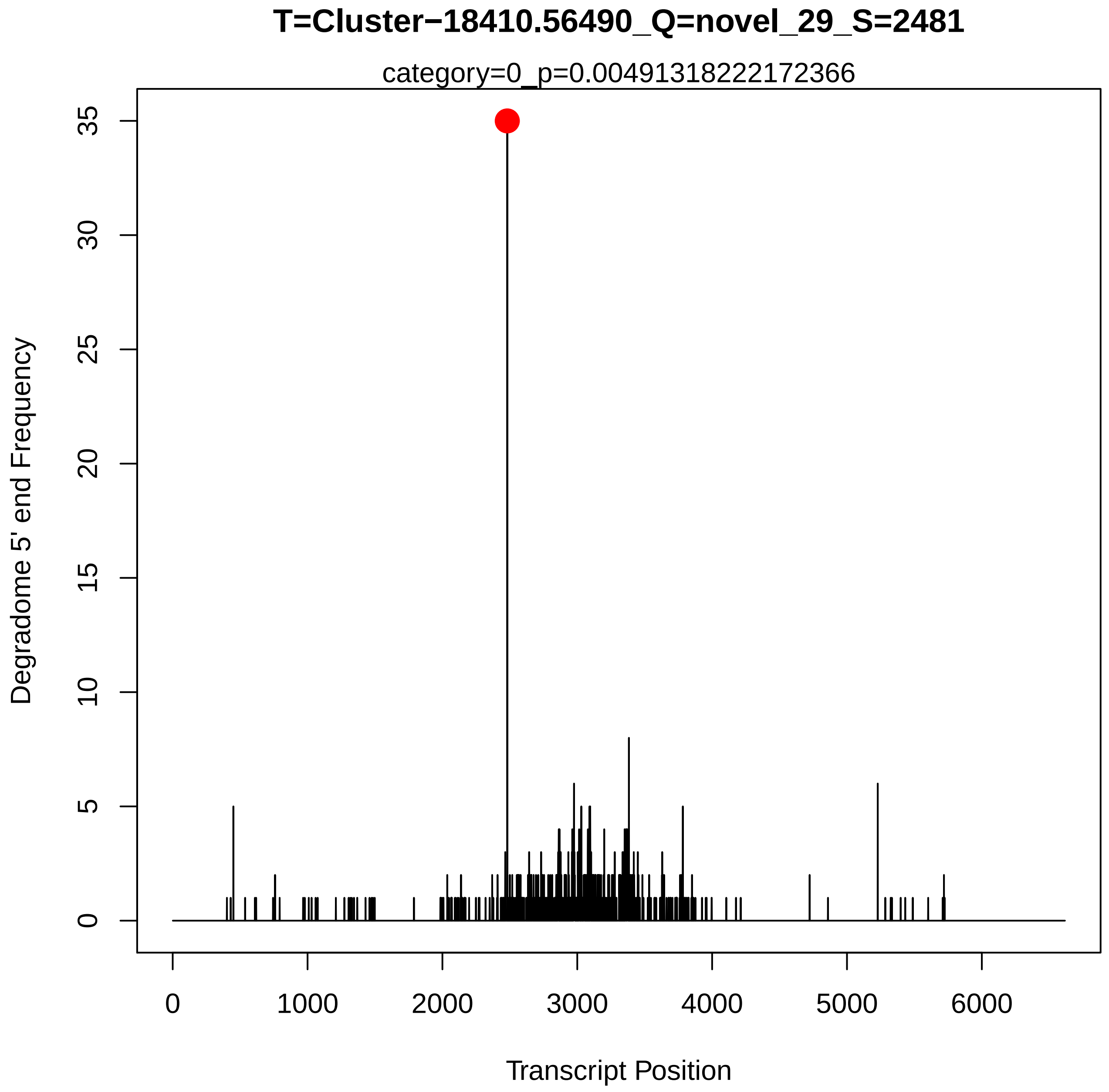
T=Cluster−18410.56490_Q=novel_29_S=2481
category=0_p=0.00491318222172366
Degradome 5' end Frequency
0
5
10
15
20
25
30
35
0
1000
2000
3000
4000
5000
6000
Transcript Position
